# Short‐duration post CT‐guided thoracic biopsy monitoring‐ clinical experience with 440 patients

**DOI:** 10.1002/jmrs.330

**Published:** 2019-03-11

**Authors:** George Asafu Adjaye Frimpong, Evans Aboagye, Pierre Amankwah, Nana E. Coleman, Nakao K. Abaidoo

**Affiliations:** ^1^ Department of Radiology Kwame Nkrumah University of Science and Technology Kumasi Ghana; ^2^ Spectra Health Imaging and Interventional Radiology Kumasi Ghana; ^3^ Department of Radiology Komfo Anokye Teaching Hospital Kumasi Ghana; ^4^ Department of Molecular Medicine Kwame Nkrumah University of Science and Technology Kumasi Ghana; ^5^ Department of Surgery Komfo Anokye Teaching Hospital Kumasi Ghana

**Keywords:** Chest imaging, CT‐guided thoracic biopsy, pneumothorax, post‐biopsy, post‐procedural monitoring

## Abstract

**Purpose:**

With several studies recording a higher percentage of complications in the first hour of post‐biopsy, this study sought to evaluate the safety in the reduction in post‐biopsy patient monitoring time after computed tomography (CT)‐guided thoracic biopsies, providing a basis for further research.

**Materials and Methods:**

This was a retrospective study involving patients who were referred to our centre for CT‐guided thoracic biopsies from January 2010 to December 2017. Patients who presented with no complications immediately after the post‐biopsy CT scan were given 30 min of post‐biopsy care after which they were discharged, and given a hot line to call in case of any complication. There was also a follow‐up call by a nurse after 24 h to inquire about any complication and general condition of the patients.

**Results:**

A total of 440 core needle thoracic biopsies were performed within the period of the study. The most common thoracic region indicated for biopsy was mediastinal (*n* = 240, 54.5%), followed by lung (*n* = 185, 42.0%). Complications were recorded at a rate of 6.4% (*n* = 28), with 4.1% (*n* = 18) been pneumothorax and pulmonary haemorrhage and haemoptysis accounting for 2.3% (*n* = 10). No relevant complications were recorded in patients who presented with no complications immediately after the post‐biopsy CT scan (*n* = 374, 85%).

**Conclusions:**

Findings from this initial study have shown that thirty minutes of post‐biopsy care could be sufficient for patients present with no complications immediately after a post‐procedural scan in CT‐guided thoracic biopsies; providing a basis for similar algorithms to be explored in a randomised control study to substantiate the observation.

## Introduction

Thoracic biopsy is a frequently performed procedure and has been associated with marked patient benefits. The advent of computed tomography (CT) and the rapid technological advancements experienced in the field over the years have made it an ideal imaging modality for the purposes of diagnosis, staging, prognostic assessment and monitoring of most thoracic pathologies, obviating surgical diagnosis.^1^, ^2^, ^3^, ^4^ As a result of the advances in imaging modalities, needle technology, immunohistochemistry and pathological techniques, clinical indications of CT‐guided thoracic biopsy have significantly changed. Currently, the list of indications includes histological diagnosis of undetermined mediastinal, chest wall and lung lesions, diagnosis of hilar lesions following negative bronchoscopy, focal parenchymal infiltrates in which an infectious organism cannot be isolated, as well as biopsy or re‐biopsy of malignancy for targeted therapy.^5^, ^6^, ^7^, ^8^, ^9^ A coaxial system is employed in thoracic biopsy, and two categories of needles are used: core‐needle biopsy (CNB) and fine‐needle aspiration biopsy (FNAB), providing specimens for histological examination and cytological examination respectively, with the CNB demonstrated to have a slightly higher overall specificity, sensitivity and accuracy.^4^, ^7^, ^8^, ^9^, ^10^, ^11^

A major concern in the performance of CT‐guided biopsies of the thoracic region is the risk of complications during and after the procedure, and the recovery time in a busy clinical setting. Potential complications associated with the procedure include pneumothorax, pulmonary haemorrhage, haemoptysis and air embolism.^12^, ^13^, ^14^, ^15^, ^16^ Some of these complications have been reported to occur within the first 2 hours, with the majority occurring in the first hour or first 24 h. As such, post‐biopsy care remains an important aspect in ensuring successful outcomes of the procedure. However, post‐biopsy care has varied considerably over the years, especially with regards to recovery time. An expert committee formed to develop guidelines for radiologically guided lung biopsy recommended a post‐biopsy care of 1 h,^17^ while a study by Dennie et al.^18^ advocated for as short as 30 min of post‐biopsy care in patients without pneumothorax. In this initial study, we report our experience with 30 min of post‐biopsy care in patients who presented with no complications after the post‐biopsy scan, providing a starting point for similar algorithms to be explored in a randomised control study to establish the observation. Derived benefits included reduction in hospital costs, patients’ early return to work and our ability to optimally utilise procedural space and ancillary staff.

## Materials and Methods

This was a retrospective study involving patients who were referred to our Interventional Radiology Clinic for CT‐guided thoracic biopsies from January 2010 to December 2017. Patients who required significant sedation or anaesthesia were excluded from the study, as these patients by default required more than 30 min of observation. Ethical approval for the study was sort from the Committee on Human Research Publication and Ethics of Kwame Nkrumah University of Science and Technology and Komfo Anokye Teaching Hospital.

### Pre‐biopsy instructions

All patients undergoing CT‐guided thoracic biopsies were instructed not to take aspirin, aspirin containing products, or non‐steroidal anti‐inflammatory medications for at least 3–5 days before the procedure. Pre‐biopsy laboratory tests were done within 2 weeks of the biopsy day (a complete blood count, prothrombin time, international normalised ratio). Standard laboratory criteria required before thoracic biopsies included platelets >50,000/mL, international normalised ratio <1.5, prothrombin time <14 sec and haemoglobin >10 mg/dL. Patients could have a light breakfast and take daily prescribed medications (excluding those mentioned above) on the morning of the procedure and are required to fast 3–4 h before the procedure. A next‐of‐kin was requested to accompany each patient for the purpose of monitoring and driving the patient home on the day of the biopsy.

### CT‐guided thoracic biopsy procedure

Patients were first taken through the process of breath holding before the procedure. A pre‐biopsy scan of the affected organ was then obtained using 64‐slice multi‐detector computed tomography scanner (Somatom Definition AS; Siemens, Erlagen, Germany), Somatom Emotion eco (16‐slice configuration, Siemens, Erlagen, Germany) and GE Light Speed VCT 64‐slice (GE healthcare, Milwaukee). This was necessary for planning the needle trajectory, optimal patient's position and direction of approach for each biopsy. Biopsies were then performed with the patient placed either supine, prone or in a lateral decubitus position to facilitate sampling of the lesion from a position closest to the body surface. Intravenous iodinated contrast (Omnipaque™ 350 mgI/mL) was administered when appropriate. Under aseptic conditions, local anaesthesia with 1% lidocaine (5–10 mL) was used. Under CT guidance, the thoracic biopsies were performed with a 16‐gauge (Gauge Size and Needle Length = 16 g × 16 cm; length of Sample Notch = 1.9 cm) BARD Coaxial system (Bard Peripheral Vascular, Inc.) for deep structures and 14‐gauge (Palium Needle, 14G × 100 mm, M.D.L. Srl‐Via Tavani 1A) for superficial and chest wall lesions. All patients underwent tissue core biopsies, in which only one puncture was made, and an average of 4–6 specimens taken with the coaxial system. None of the mediastinal biopsies required trans‐pulmonary approach. The core tissues were then placed in a sterile formalin‐filled container and sent for histological evaluation.

### Post‐biopsy care

Post‐biopsy CT scan was performed immediately for all the cases using contrast. Patients without post‐biopsy complications were made to lie down for 30 min in a lateral decubitus position. During this time, patients’ blood pressure and pulse were checked every 10 min, and also analgesics and/or antibiotics were given depending on patient's pain level or risk factors respectively. Patients without post‐biopsy complications were discharged after 30 min, and given a hot line to call in case of any complications. There was a follow‐up call by a nurse after 24 h to inquire about any complications and the general condition of the patient. During the follow‐up call, patients were asked whether they had experienced delayed symptoms such as worsening pain, generalised discomfort and shortness of breath.

**Figure 1:**
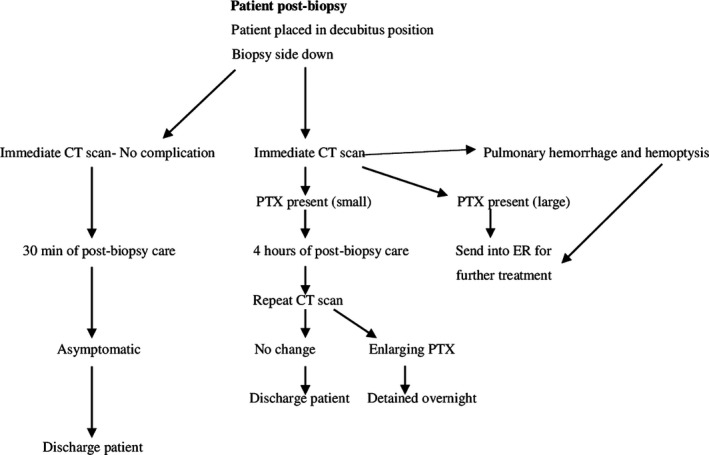
Flow chart showing the proposed patient treatment algorithm after CT‐guided thoracic biopsy. PTX = pneumothorax; ER = emergency room.

A flow chart showing the proposed patient treatment algorithm after CT‐guided thoracic biopsy is shown in Figure 1.

## Results

A total of 440 patients who were referred for CT‐guided thoracic biopsies within the study period were included. The mean age of the patients was 52.2 ± 18.3 years, with the maximum and minimum age being 83 and 8 years respectively. A greater percentage of the patients were males (*n* = 264, 60.0%), with a female population of 40.0% (*n* = 176). Thoracic regions indicated for CT‐guided biopsies within the period of study included mediastinal, lung, pleural and chest wall. The mean lesion diameter was 8.7 cm, with a range of 1.2–18.4 cm. Two hundred and sixty‐six of the lesions on which biopsies were performed had a diameter ranging from 4.0 to 10.0 cm, whereas 28 were less than 4 cm. The 16‐guage BARD Coaxial system and 14‐gauge Palium were used in 315 and 125 patients respectively. The distribution is shown in Table 1.

**Table 1:** Characteristics of the study population and complications

Characteristics	Study population (*n* = 440)	Presence of complication (*n* = 28)	Absence of complication (*n* = 412)
Gender			
Male	264	21	243
Female	176	7	169
Thoracic region			
Mediastinal	240	0	240
Lung	185	26	159
Pleural	8	2	6
Chest wall	7	0	7
Lesion diameter			
<4 cm	28	10	18
4–10 cm	266	15	251
>10 cm	146	3	143
Needle size			
14‐gauge	125	3	122
16‐gauge	315	25	290
Post‐biopsy complications			
Pneumothorax	18		
Pulmonary haemorrhage and haemoptysis	10		
None	412		
Reported delayed symptoms			
Worsening pain	88		
Generalised discomfort	22		
Shortness of breath	13		
None	317		

Post‐biopsy complications were recorded at a rate of 6.4% (*n* = 28), with 4.1% (*n* = 18) been pneumothorax and pulmonary haemorrhage and haemoptysis accounting for 2.3% (*n* = 10). The complications were recorded only in the lung (*n* = 26) and pleural (*n* = 2) biopsies. No relevant complications were recorded in those who presented with no post‐biopsy complications (*n* = 412) on follow‐up, except for worsening pain (*n* = 88), generalised discomfort (*n* = 22) and shortness of breath (*n* = 13). Also, no contrast allergies and fatalities were recorded during the entire duration of the study.

## Discussion

Optimal utilisation of procedural space and ancillary staff is a key to the successful operation of any outpatient health care facility. This is particularly true in cases involving post‐procedural care such as CT‐guided biopsies. As such, recommendations ranging from 30 min to 1 h of post biopsy care have been proposed by various studies over the years.^17^, ^18^ This was a retrospective study which evaluates the safety in the reduction in post‐biopsy patient monitoring time to 30 min, in patients who presented with no post‐biopsy complications immediately after CT‐guided thoracic biopsies in an outpatient Interventional Radiology Clinic, providing a basis for similar algorithms to be explored in a randomised control study to substantiate the observation. The novelty of this work as compared to other studies especially that of Dennie et al.,^18^ whom we duly acknowledge for proposing 30 min of post‐biopsy care, is that we report on only core‐needle biopsies while they reported on mostly on fine‐needle aspiration biopsies (approximately 87% of the cases), in which complications are less likely to occur compared to the former. Also, we employed CT scan, which is the gold standard for evaluating lung complications, while they employed fluoroscopic guidance in majority of the cases (98.4%).

The study involved 440 patients, out of which 412 (93.6%), who presented with no post‐biopsy complications were given a post‐biopsy care of 30 min. The patients were eventually discharged as no relevant complication was identified during this observational period. Also, no relevant delayed complication was recorded during the follow‐up period. However, 123 (29.9%) out of the 412 patients reported delayed symptoms such as worsening pain, generalised discomfort and shortness of breath, which are usually associated with the procedure. This was contrary to that reported by Dennie et al^18^ in their study which also recommended 30 min of post‐biopsy care in patients who presented with no pneumothorax. The work which mostly involved fine‐needle aspiration biopsy (FNAB), recorded 7 patients who developed a symptomatic pneumothorax after discharge. This difference is probably due to the different imaging modalities utilised. Fluoroscopic guidance which was employed in majority of the cases of Dennie et al^18^ can miss very small pneumothorax which will be evident in CT guidance which was employed in this study. Also, despite the difference in lung biopsy cases (321 less in our case) which may have contributed to the disparity, the core‐needle biopsy employed in this study posed significant risks compared to the FNAB utilised in their study.

The majority of the biopsies were performed on lesions involving the mediastinum, followed by lung, and were mostly larger lesions as shown in Table 1. Lung lesions are generally uncommon in our geographical region as the rate of smoking is very low due to socio‐cultural practices. Larger lesions are however usually seen in our geographic region as most malignancies are diagnosed at advanced stages primarily due to paucity of diagnostic centres and socio‐cultural and economic factors. The larger lesions coupled with technological advancements in the field, probably played a significant role in the absence of delayed complications observed in our study population. Smaller lesions, by virtue of their size may require multiple attempts to target the lesions precisely, thus more likely to be associated with the development of pneumothorax and other complications.^18^ This finding is reinforced by several studies which have reported that an increased rate of pneumothorax correlated with smaller lesion size in patients.^19^, ^20^, ^21^

Improved image guidance and the use of smaller gauge needles have resulted in significantly lowered rate of complications and enhanced safety.^16^ Pneumothorax and pulmonary haemorrhage and haemoptysis were the only complications recorded in this study, at a combined rate of 6.4%, and all the complications were associated with only lung (*n* = 26) and pleura biopsies (*n* = 2). Pneumothorax was the most prevalent complications and it occurred in both lung (*n* = 16) and pleura (*n* = 2) biopsies. Other complications reported in literature include haemoptysis,^15^ air embolism^16^ and nerve injury.^22^ As noted earlier, the 412 patients who were discharged after 30 min reported no relevant delayed complications on follow‐up. This suggests that patients in our study had very little chance of developing a relevant complication once none was detected in a post‐biopsy CT scan.

The study was however limited by the fact that subsequent evaluation of the patients was mainly based on telephone conversation.

## Conclusions

This initial study has shown that 30 minutes of post‐biopsy care could be sufficient for patients who present with no complications after a post‐procedural scan in CT‐guided thoracic biopsies, providing a starting point for similar algorithms to be explored in a randomised control study to establish the observation. This approach has the potential to significantly reduce medical costs and enable optimal utilisation of procedural space and ancillary staff. The algorithm proposed in this study however appears to be more appropriate for larger lesions, and that further studies are needed to evaluate its suitability for smaller lesions.
